# From H1N1 to COVID-19: What we have seen in children with hemoglobinopathies

**DOI:** 10.1016/j.clinsp.2021.100004

**Published:** 2022-01-29

**Authors:** Claudia de Melo Oliveira, Victor Jablonski Soares, Ciliana Rechenmacher, Liane Esteves Daudt, Mariana Bohns Michalowski

**Affiliations:** a Programa de Pós-Graduação em Saúde da Criança e do Adolescente, Departamento de Pediatria, Universidade Federal do Rio Grande do Sul, Porto Alegre, Brazil; b Faculdade de Medicina, Universidade Federal do Rio Grande do Sul, Porto Alegre, RS, Brazil; c Laboratório de Pediatria Translacional, Serviço de Pesquisa Experimental, Hospital de Clínicas de Porto Alegre, Porto Alegre, RS, Brazil; d Unidade de Hematologia e Transplante de Medula Óssea Pediátrica, Serviço de Hematologia Clínica, Hospital de Clínicas de Porto Alegre, Porto Alegre, RS, Brazil; e Serviço de Oncologia Pediátrica, Hospital de Clínicas de Porto Alegre, Porto Alegre, RS, Brazil

**Keywords:** SARS-CoV-2, Hemoglobinopathies, Child, Virus diseases, Pandemics

## Abstract

This work aimed to better understand the impact of pandemics of respiratory viruses on children with hemoglobinopathies through a comprehensive review of the literature. MEDLINE, SCIELO, LILACS, and PUBMED were used as data sources to find articles without time period restrictions. Previous observations suggest that patients with hemoglobinopathies are a group especially susceptible to the complications of viral respiratory infections, with greater morbidity and mortality related to them. Within this context, this review found that, during the 2009 H1N1 pandemic, the risk of hospitalization in children and adults increased, especially in patients with a history of complications such as acute chest syndrome. In addition, the Coronavirus Disease 2019 (COVID-19) pandemic appears to have less repercussion among children with hemoglobinopathies compared to adults, similar to what is seen in the general population. In the H1N1 pandemic, patients with hemoglobinopathies behaved as a group more susceptible to complications, with increased morbidity and mortality. However, for COVID-19, the existing data to date on these patients do not show the same clinical impact. Thus, although these children deserve attention in case of infection due to their potential risks, they seem to have a favorable evolution.

## Highlights

• Children with hemoglobinopathies have less severe conditions with Coronavirus 2019 Disease (COVID-19) compared to adults, which is similar to that observed in the general population
• In the H1N1 pandemic, patients with hemoglobinopathies behaved as the group most susceptible to complications, with increased morbidity and mortality

## Introduction

In December 2019, a new coronavirus was identified by Chinese scientists in the city of Wuhan in Hubei, China, after studying patients with pneumonia of unknown origin.^1^ The virus called Severe Acute Respiratory Syndrome Coronavirus 2 (SARS-CoV-2) spread quickly to other countries, challenging the economic, medical, and public health infrastructures.^2^ Existing studies have already shown that all age groups are susceptible to SARS-CoV-2 and that transmission occurs through oral fluid droplets. The spectrum of the disease varies from asymptomatic patients to cases in which the infection, named Coronavirus Disease 2019 (COVID-19), causes symptoms such as fever, cough, sore throat, fatigue, headache, and in more severe cases, acute respiratory syndrome, and multiple organ dysfunction.^3^

Hemoglobinopathies are a group of high prevalence diseases. With approximately 7% of the worldwide population being carriers, hemoglobinopathies are the most common monogenic diseases and one of the world's major health problems.^4^ In this context, these patients have been impacted not only by COVID-19 but also by other pandemics of respiratory viruses that have affected the human population in recent decades, such as Influenza A (H1N1), in 2009. Considering that comorbid chronic diseases are strongly correlated with disease severity among COVID-19 patients,^5^ those with hemoglobinopathies could be at higher risk of worse prognosis, especially in the pediatric population. In this review, the authors describe the characteristics that make children with hemoglobinopathies a group that requires special attention and the already known impact of respiratory viruses on this population. The following electronic databases were used: MEDLINE, SCIELO, LILACS, and PUBMED.

## Overview of hemoglobinopathies

Hemoglobinopathies are genetic disorders that affect hemoglobin molecules, a protein present in red blood cells. Most of the hemoglobinopathies result from mutations that alter the amino acid sequence of a globin chain, thus, changing the physiologic properties of the variant hemoglobin and producing the characteristic clinical abnormalities. Thalassemia syndromes, on the other hand, arise from mutations that impair the production or translation of globin mRNA, leading to deficient globin chain biosynthesis.^6^

In relation to hereditary hemoglobinopathies, the most common are hemoglobin S, hemoglobin C, beta-thalassemia, hemoglobin E, and alpha thalassemia.^4^ Sickle cell disease (SCD) is characterized by alteration of the two beta-globin genes, where at least one is an S gene. These sickle cells are more rigid and, therefore, can lead to pain, organ damage, and acute chest syndrome (ACS). These are the main known reasons for increased general morbidity and mortality in this patient population.

A hemoglobin disorders epidemiology study of 2008 estimates that 1.1% of couples in the world are at risk of having children with hemoglobinopathies, and 2.7 of every 1000 pregnancies are affected, with birthplace having a big impact on prognosis. If affected, children who are born in high-income countries have long-term survival with a chronic disorder, while the majority of those born in poor countries die before the age of five. Hemoglobinopathies are responsible for 3.4% of mortality in children younger than five years old around the world and for 6.4% in Africa.^7^ Approximately 1000 children with SCD are born in Brazil annually.^8^

Two retrospective studies described data and characteristics of SCD in Brazil and its mortality. Fernandes et al.^9^ studied 1,396 children that had a hemoglobin profile compatible with SCD, collected in the Neonatal Screening Program in Minas Gerais. Among these cases, 78 patients died between 1998 and 2005; most of them (71.8%) were children younger than two years old. The main causes of death were infections (38.5%) and Acute Splenic Sequestration (16.6%). In another study, in-hospital mortality rates were described, and differences were observed between the states of the country: 2.0% in Bahia, 2.1% in Rio de Janeiro, and 1.0% in São Paulo. Moreover, mortality was approximately five times higher in adults than in children and adolescents. Variations in lethality probably reflect social differences between Brazil and other countries, as well as the regions in the country.^10^ Another retrospective study analyzed the mortality rate in 1,676 patients who had SCD and b- thalassemia from 1998 to 2012 at HEMORIO where 281 patients died with a mortality rate of 10.8% for children (< 18 years old) and 18.87% for adults. The life expectancy was 21.3 years inferior to the general Brazilian population.^11^ Knowledge of these baseline data is important because it allows for the assessment of the impact of recent respiratory virus pandemics, such as H1N1 and COVID-19, on the survival of these patients.

## H1N1 pandemic: what we learned

The first reported cases of H1N1 appeared in Mexico in February 2009. In April of the same year, the WHO declared H1N1 a public health emergency of global importance, and in June, when there had been reported more than 26,000 cases in 73 countries, the infection was considered a pandemic.^12^ In a year and a half, the total number of deaths worldwide was approximately 20,000, and more than 214 countries were affected.^13^ This pandemic also had a significant impact on Brazil. The cumulative incidence in the country in 2009 was 28/100,000 inhabitants, with 52,827 confirmed cases. The case fatality rate reported by Rosseto and Luna was 3.9% (2,056/52,827) in 2009 and 12.4% (120/970) in 2010. Nevertheless, the authors called attention to the fact that the case-fatality rate was calculated using different populations, times, and places, creating biases in possible comparisons between the two periods. Moreover, they described limitations due to the quality of data collection, in addition to underreporting and lack of a prioritized record-keeping system for serious cases or those from risk groups.^14^

SCD was one of the comorbidities reported among patients hospitalized during the H1N1 pandemic as they have a higher predisposition to organ complications and hypoxia.^15^ 2,200 children with SCD were evaluated in London from April to August 2009. According to this survey, approximately 40 children with SCD were diagnosed with H1N1, 50% of them were admitted to the hospital, and 25% developed acute chest syndrome. The hospitalization rate was considered higher compared to the general population, which was 7%.^16^ Another study showed that patients with hemoglobinopathies, in general, had approximately three times more chances of being hospitalized.^17^ A retrospective cohort conducted by Johns Hopkins Hospital analyzed cases of influenza A, B, and H1N1 in children and young adults with SCD. It was found that those with H1N1 tended to be older, have asthma, develop ACS more frequently, and present more severe pain.^18^ In addition, these patients needed intensive care more often, receiving more blood transfusions and antiviral treatments.

In another retrospective study, 48 patients with SCD diagnosed with H1N1 at the Cincinnati Children's Hospital Medical Center were evaluated.^19^ Among these, 23 (48%) patients had to be hospitalized, 10 of which were due to ACS. No patient needed mechanical ventilation or died. History of ACS and asthma were associated with higher rates of hospitalization and the likelihood of developing a new ACS. Furthermore, Colombatti et al.^20^ conducted a retrospective study with 11 Italian Pediatric Hematology-Oncology Units, including data from 17 patients that were admitted for laboratory-confirmed H1N1 infection among 322 patients with SCD. Half of them had ACS and flu-like syndrome with vaso-occlusive crises. Also, 41% of the patients received a simple transfusion due to a fall in hemoglobin, while 29% underwent erythrocytapheresis.

Thus, the H1N1 pandemic showed that patients with SCD are especially susceptible to the complications of viral respiratory infections and have increased morbidity. Within this group, patients with a previous history of ACS and bronchial hyperreactivity were especially likely to need hospitalization and develop ACS.^19^ Even though these studies did not address any deaths by H1N1 in patients with SCD, critical illness was especially common in older patients compared to the children population.^18^ The main studies on H1N1 and hemoglobinopathies and their results are described in Table 1.

**Table 1:** Data from articles reporting H1N1 infection in patients with hemoglobinopathies.

Author, year	Design	Study population	Main findings
Strouse et al.,^18^ 2010	Retrospective cohort	123 SCD patients < 22 yo with diagnoses of influenza A (66); B (28); and H1N1 (29)	Patients with seasonal influenza were younger and less likely to have asthma. Patients with H1N1 more often developed ACS, severe pain, and illness requiring intensive care. The most frequent treatments used with these patients were antiviral agents, red blood cell transfusions, and exchange transfusions (10% for those with H1N1 vs. 3% for seasonal influenza, p=0.045). No patient died.
George et al.,^19^ 2011	Retrospective chart review	48 children with all forms of SCD and diagnosis of H1N1 or influenza A	The most common diagnosis in hospitalized patients was ACS. No mechanical ventilation was required, or death reported. History of ACS or reactive airway disease was correlated with a higher rate of admission and of ACS incidence during the acute illness.
Colombatti et al.,^20^ 2011	Retrospective survey	17 patients < 17 yo with SCD and H1N1	15 HbS/HbS and 2 HbS/betha patients. Mean age: 7 years (range 1–17). 8 (47%) patients had ACS; 8 (47%), flu-like syndrome with vaso-occlusive crises; and 1 (6%), acute splenic sequestration. 7 (41%) patients received a simple transfusion and 5 (29%) underwent erythrocytapheresis.

SCD, Sickle Cell Disease; yo, years old; ACS, Acute Chest Syndrome.

## SARS-CoV-2 pandemic: what we know so far

According to the WHO's international registry, on May 5^th^, 2021, countries on all six continents had reported more than 150 million cases of COVID-19 and over 3.2 million deaths.^21^ The Center for Disease Control COVID-19 Response Team has reported that, as of April 2^nd^, 2020, only 1.7% of the COVID-19 cases reported occurred in pediatric patients, despite 22% of the US population being younger than 18 years old. It also estimated that 20% of these pediatric patients were hospitalized, compared to 33% adults aged 18–64 years.^22^ Considering the previous experience of vulnerability of children with hemoglobinopathies during the H1N1 pandemic, a significant increase in the morbidity and mortality of these patients during SARS-CoV-2 infection was also expected.^23^ The main studies on SARS-CoV-2 and children and their findings are summarized in Table 2.

**Table 2:** Data from reported cases of SARS-CoV-2 infection in patients with hemoglobinopathies.

Author, year	Design	Study population	Main findings
Telfer et al.,^25^ 2020	Real-time survey	195 patients (17 children) with confirmed and suspected diagnoses of COVID-19 and hemoglobinopathy or rare inherited anemia	166 patients had SCD; 26, thalassemia; and 3, rare inherited anemia. None required non-invasive and/or mechanical ventilation. No death was reported in this group.
Singh et al.,^26^ 2021	Retrospective cohort	624 black patients with diagnoses of COVID-19, 312 with SCD/SCT and 312 without SCD/SCT trait	Patients with SCD had a higher risk of hospitalization (RR = 2.0) and development of pneumonia (RR = 2.4) and pain (RR = 3.4) compared with those without SCD/SCT. The fatality rate was not significantly different. No comparison between adults and children was made.
Minniti et al.,^28^ 2021	Cohort	66 patients with SCD and diagnosis of COVID-19	Patients aged > 50 years, with preexisting cardiopulmonary, renal disease, and/or stroke who were not receiving hydroxyurea had as risk factors for death high serum creatinine, lactate dehydrogenase, and D-dimer levels, irrespective of genotype or sex. 75% required hospitalization, and 10.6% died. No children died.

SCD, Sickle Cell Disease; RR, Relative Risk; SCT, Sickle Cell Trait.

The Medical College of Wisconsin established the SECURE-SCD Registry to collect data on COVID-19 cases occurring globally in patients with SCD.^24^ Professionals were asked to report only COVID-19 confirmed cases and to do it only after resolution of acute illness or death. Until April 30^th^, there were 784 registered cases. The mean age was 22.44 years old, with almost half of the patients being younger than 19 (48.1%). Of the total, 137 were asymptomatic, 431 had mild symptoms, 105 had moderate clinical severity, while 89 and 19 patients had severe and critical forms of the disease, respectively. Even though no statistical analysis of this data has been provided by the SECURE-SCD Registry hitherto, from a total of 19 deaths, only one was recorded on the < 19 years old group. Also, it is noteworthy that previous hospitalization for pain (equal to 3 or more in the last three years) was a common characteristic both in the total group (40.1%) and in those who died (68.4%).

Similar results were found in a real-time national survey on COVID-19 in hemoglobinopathy and rare inherited anemia patients.^25^ Sickle cell disease accounted for 166 of the 195 cases reported. There were 149 adults and 17 children (defined as ≤ 18 years). One hundred and twenty-eight (77.1%) SCD patients were admitted to the hospital, of whom 15 (11.7%), all adults, required non-invasive and/or mechanical ventilation. The outcomes of 142 patients with SCD with a completed course of infection were analyzed. A total of 131 (92.2%) recovered, while 11 (8.4%) died. The median age of patients who died was higher (51 years, ranging from 19 to 68 years) than those who recovered (31 years, ranging from 6 weeks to 72 years, p = 0.0042). No deaths occurred in children. The association of patient variables with survival was analyzed in SCD patients (n = 77) who were admitted to the hospital with a laboratory-confirmed COVID-19 diagnosis. The overall mortality in the group was 10.4%.

In contrast to the association of older age and male sex with the risk of COVID-19 related death in other populations, no significant correlation was found with age, sex, or SCD genotype. Mortality was higher in females and mild genotypes, although the differences were not significant. Patients with thalassemia accounted for 26 of the cohort. Two deaths were reported in transfusion-dependent patients 92y = HbH (cancer and splenectomy) and 53y = TDT (iron overload and diabetes); both had concurrent morbidities. Only three patients with rare inherited anemia (unstable hemoglobin Hb Köln: 2; hereditary elliptocytosis: 1) were reported. All received transfusion for acute hemolysis during the COVID-19 episode and subsequently recovered. Considering the presumed higher risk of infection for the patients with hemoglobinopathies, data on the hospitalization rate of these patients is also an important parameter. A retrospective cohort conducted by Singh et al.^26^ collected data to compare COVID-19 outcomes between individuals with SCD and sickle cell trait (SCT) and those without these diseases. The authors found that patients with COVID-19 and SCD remained at a higher risk of hospitalization (Relative Risk [RR=2.0]; 95% CI, 1.5–2.7) and development of pneumonia (RR=2.4; 95% CI, 1.6–3.4) and pain (RR=3.4; 95% CI, 2.5–4.8) compared with the group who do not have SCD or trait. On the other hand, the case fatality rates were not significantly different. There were also no significant differences in COVID-19 outcomes between individuals with SCT and control. This suggests that heterozygous patients have a different risk when affected by COVID-19 when compared to homozygous SCD. Although the age of patients was disregarded in this study, a different investigation found that young adults with SCD had a lower risk of intensive care unit admission when compared to older inpatients,^27^ but it lacks statistical power.

In another cohort, Minniti et al.^28^ analyzed 66 patients with SCD and COVID-19, of which nine were children, from five academic centers in the United States. In their study, it was possible to differentiate the characteristics of patients who were hospitalized from those who were not. There was no difference in SCD genotype or age among those who needed admission. However, the presence of chronic kidney disease and higher leukocyte count were associated with the need for hospitalization, while the use of SCD-modifying therapy was more common in outpatients. Among the seven deaths recorded in this study, none occurred in the pediatric age group. Overall, these studies suggest that patients with SCD are at higher risk of hospitalization due to COVID-19, especially those with previous comorbidities such as chronic kidney disease and of developing complications during the hospital stay in comparison with those without hemoglobinopathies.

Regarding thalassemia, most of the literature so far does not deal so much with prognosis and clinical impact but with infection rates. Nevertheless, with the course of the SARS-CoV-2 pandemic and epidemiological observations, the issue of a possible protective mechanism for patients with hemoglobinopathies arose. Lansiaux et al.^29^ used multiple linear regression to observe the population of three Italian regions with a higher prevalence of heterozygosity for thalassemia and lower infection rates to formulate the hypothesis of immunity. However, the very limitations inherent in this study make the causal relationship questionable.

Furthermore, in a multicenter, retrospective, and cross-sectional study, Karimi et al.^30^ estimated the prevalence of COVID-19 in the general Iranian population. In April 2020, it was 11.01/10,000 inhabitants, while for the population with thalassemia, it was 8.17/10,000 (p = 0.24). Mortality was significantly higher in the latter group (26.6%) compared to the former (6.34%) (p = 0.001). In another study,^31^ a cohort of 182 patients with SARS-CoV-2 infection was analyzed. Nineteen had the genetic trait for beta-thalassemia, and none were in the group of severely ill patients. However, the molecular mechanism that supports that hypothesis of a possible protective mechanism, based on the SARS-CoV-2 attack on the 1-beta chain of hemoglobin, was rejected.^32^ Other studies also do not correlate the increased severity of SARS-CoV-2 infection in patients with thalassemia syndromes.^33^ Given the weak evidence, further research is needed to estimate a possible correlation between thalassemia and the COVID-19 infection rate.

## Conclusion

The H1N1 and SARS-CoV-2 pandemics are recent in our history and have severely affected humankind, causing numerous deaths. Despite the effort of the scientific community, these viruses’ complete molecular mechanisms of action, as well as how they affect different populations, are still unclear. Thus, these understandings may help to minimize the impact and the number of deaths. Therefore, this literature review aimed to contribute to the care of children with hemoglobinopathies in the context of COVID-19.

Although there was great concern with patients with hemoglobinopathies in the COVID-19 pandemic due to the complications observed during the H1N1 pandemic, this population was not so severely affected. Moreover, the gathered data indicates that children with hemoglobin disorders have fewer symptoms and better outcomes when compared to adults. This finding is similar to that found by other reviews^34^ and could be related to the same protective mechanisms hypothesized for the general pediatric population.^35^ Nonetheless, some studies indicate that, as in the first pandemic, both adults and children with hemoglobinopathies may be at higher risk of hospitalization and intrahospital complications. Those with comorbidities, like chronic kidney disease, could be at an even higher risk. These data, however, should be looked at carefully since this is a heterogeneous group of diseases with different pathophysiologies in which the interaction with SARS-CoV-2 could result in different outcomes.

## Declaration of Competing Interest

The authors declare no conflicts of interest.
